# Somatotopy of the sensory thalamus: inputs from directional deep brain stimulation in pain patients

**DOI:** 10.1002/acn3.52067

**Published:** 2024-04-26

**Authors:** Aurelie Leplus, Petru Isan, Anne Balossier, Sarah Mouffok, Anne Donnet, Theodore Papadopoulo, Michel Lanteri‐Minet, Jean Regis, Denys Fontaine

**Affiliations:** ^1^ Department of Neurosurgery, FHU INOVPAIN, CHU de Nice Université Côte d'Azur Nice France; ^2^ UR2CA Université Côte d'Azur Nice France; ^3^ Department of Neurosurgery Hopital La Timone, APHM, FHU INOVPAIN Marseille France; ^4^ INRIA Center Université Cote d'Azur Sophia Antipolis France; ^5^ Pain Clinic Hopital La Timone, APHM Marseille France; ^6^ Neuro‐Dol, Trigeminal Pain INSERM/UCA, U1107 Clermont‐Ferrand France; ^7^ Pain Clinic, FHU INOVPAIN, CHU de Nice Université Côte d'Azur Nice France

## Abstract

**Objective:**

The sensory ventroposterior (VP) thalamic nuclei display a mediolateral somatotopic organization (respectively head, arm, and leg). We studied this somatotopy using directional VP deep brain stimulation (DBS) in patients treated for chronic neuropathic pain.

**Methods:**

Six patients with central (four) or peripheral (two) neuropathic pain were treated by VP DBS using directional leads in a prospective study (clinicaltrials.gov NCT03399942). Lead‐DBS toolbox was used for leads localization, visualization, and modeling of the volume of tissue activated (VTA). Stimulation was delivered in each direction, 1 month after surgery and correlated to the location of stimulation‐induced paresthesias. The somatotopy was modeled by correlating the respective locations of paresthesias and VTAs. We recorded 48 distinct paresthesia maps corresponding to 48 VTAs (including 36 related to directional stimulation).

**Results:**

We observed that, in each patient, respective body representations of the trunk, upper limb, lower limb, and head were closely located around the lead. These representations differed across patients, did not follow a common organization and were not concordant with the previously described somatotopic organization of the sensory thalamus.

**Interpretation:**

Thalamic reorganization has been reported in chronic pain patients compared to non‐pain patients operated for movement disorders in previous studies using intraoperative recordings and micro‐stimulation. Using a different methodology, namely 3D representation of the VTA by the directional postoperative stimulation through a stationary electrode, our study brings additional arguments in favor of a reorganization of the VP thalamic somatotopy in patients suffering from chronic neuropathic pain of central or peripheral origin.

## Introduction

The somatosensory thalamic nuclei, namely the ventral posterior nuclei,^1^ are a major relay on the somatosensory pathways. Deep brain stimulation (DBS) of the thalamic sensory nuclei has been proposed since the 1960s to treat medically refractory neuropathic pain^2^, ^3^, ^4^ with long‐term success varying from 30% to 80% across series.^5^ Within the sensory thalamic nuclei, it is recommended to follow the somatotopy to implant the DBS lead according to the pain location that is, targeting the ventral posterior medial nucleus (VPM) for facial pain or the ventral posterior lateral (VPL) nucleus for limb pain.^6^

**Figure 1:**
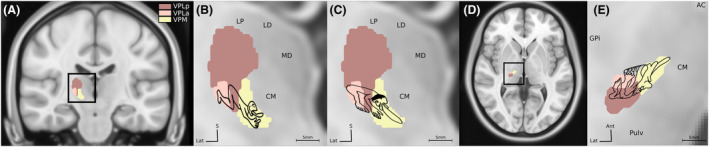
Schematic representation of the human sensory thalamus somatotopic organization overlaid on the right sensory thalamic nuclei on a coronal (A) and axial (D) views: (B) as proposed by Ohye et al.^9^ (C) by Hassler and Walker^10^ and by Guiot et al.^13^ in an oblique axial plane (E). (A) coronal and (D) axial views of the human sensory thalamic nuclei: ventro‐postero‐lateral posterior (VPLp—brown), ventro‐postero‐lateral anterior (VPLa—pale brown), and ventro‐postero‐medial (VPM—yellow) nuclei as defined in the Morel atlas.^29^ AC, anterior commissure; CM, centro median nucleus; GPi, globus pallidus internus; LD, lateral dorsal nucleus; LP, lateral posterior thalamic nucleus; MD, medial dorsal nucleus; Pulv, pulvinar.

Indeed, as for the primary sensory cortex, the sensory thalamic nuclei are somatotopically organized, as reported in animal and human electrophysiological studies. In monkeys, studies of the thalamic receptive fields (RF), namely the body areas whose physical stimulation elicits thalamic electrophysiological recording changes, showed that these nuclei were organized according to a mediolateral gradient. The face and hand RF were located more medially and the trunk, foot, and tail RF more laterally.^7^, ^8^ In humans, several studies, conducted in patients treated by thalamotomy or DBS of the ventral intermediate nucleus (VIM) of the thalamus for disabling tremor,^9^, ^10^, ^11^, ^12^, ^13^ have also reported a sensory thalamic somatotopic organization. Intraoperative microelectrode recordings were used to differentiate the VIM from the VPL/VPM, just posterior to it, and additionally allowed to study the projected fields (PF), that is, body areas where paresthesias were induced by stimulation of the thalamic electrode. Hassler and Walker,^10^ then Ohye et al.^9^ studied thalamic RF and PF in patients with movement disorders and described a mediolateral somatotopy (Fig. 1). The face was represented medially, the lower limb laterally and the upper limb between both. In addition to a mediolateral organization, Guiot et al.^13^ described an anteroposterior organization; the trunk represented posteriorly and the distal part of the limbs being more anterior (Fig. 1). Exploring 140 patients with movement disorders and 33 with intractable pain, Tasker^11^ and Lenz et al.^12^, ^14^ observed similar results but noted a significant interindividual variability.

In these studies, despite minor variations, a mediolateral somatotopy appeared to be the rule, with RF and PF seemingly matching.^11^, ^12^, ^15^, ^16^ However, a few studies performed in patients suffering from chronic neuropathic pain resulting from central deafferentations reported that this thalamic organization differed from non‐pain patients, with a RFs‐PFs mismatch.^12^, ^16^

Since the 1970s, thalamic DBS for pain has become far less frequent and DBS hardware has improved, especially with the development of directional leads.^17^, ^18^ Directional DBS leads allow to stimulate brain tissue in one single direction around the electrode to better refine the stimulated area, also called the volume of tissue activated (VTA), offering the opportunity to study the effects of the stimulation of distinct VTAs around a single electrode.

The aim of the present study was to study the somatotopy of the sensory thalamus in patients suffering from chronic neuropathic pain, by analyzing the paresthesias induced by directional thalamic DBS according to the location of the VTAs.

## Methods

### Patients

All the patients were included in a prospective study exploring combined thalamic and anterior cingulate gyrus (ACC) DBS in chronic pain patients (EMOPAIN study, clinicaltrials.gov NCT03399942), whose protocol has been previously published.^19^ We included six patients suffering from chronic (duration >1 year), severe (visual analogue scale (VAS) score > 6/10), with high emotional impact (Hospital Anxiety and Depression (HAD) score sub‐scores > 10), unilateral neuropathic pain, considered resistant to drugs recommended for neuropathic pain at sufficient doses and durations (including at least one antiepileptic and one antidepressant). Noninclusion criteria were cognitive impairment (MMSE score < 24), DSM‐IV axis I psychiatric comorbidities, and contraindications to surgery, DBS, anesthesia, or MRI. Demographic data and characteristics of pain are detailed in Table 1. All the patients were treated with combined bilateral ACC—unilateral thalamus DBS with the aim to alleviate refractory pain. Description of the somatotopy of the sensory thalamus was performed as an ancillary study, based upon the DBS induced subjective paresthesias in the postoperative period.

**Table 1:** Characteristics of six patients with neuropathic pain, pain locations, and pain mechanisms.

	Sex	Age	Location of the lesion	Pain location	Pain side	DBS target	Pain duration (years)	Mechanism of pain	VAS pre‐op	HAD A pre‐op	HAD D pre‐op
Patient 1	F	50	Lenticular nucleus, internal capsule	UL, Face	R	VPM	3	Central neuropathic pain: post hemorrhagic stroke	6	12	14
Patient 2	M	42	Sciatic nerve trunk	LL	L	VPL	7	Peripheral pain: sciatic nerve lesion	6	11	4
Patient 3	M	47	Mesencephalic	Hemi‐body (LL predominant)	R	VPL	5	Central neuropathic pain: mesencephalic cavernoma	7	10	10
Patient 4	M	42	Fronto‐parieto‐insular regions	Hemi‐body (UL and LL predominant)	L	VPL	5	Central neuropathic pain, middle cerebral artery ischemic stroke	7	11	14
Patient 5	F	48	Trigeminal nerve	Face	R	VPM	4	Trigeminal nerve lesion	8	10	8
Patient 6	M	69	Cervical spinal cord	UL, LL	R	VPL	2.5	Spinal cord injury	8	13	13

HAD, Hospital Anxiety and Depression; LL, lower limb; UL, upper limb; VAS, visual analogue scale; VPL, ventro‐postero‐lateral nucleus; VPM, ventro‐postero‐medial nucleus.

### Ethical considerations

The study has been approved by an Ethical Committee (Comité de Protection des Personnes Sud Mediterranée, 2017; N° IDRCB: 2017‐A00032‐51). Informed consent was provided by all patients before inclusion.

### Surgical procedure and trajectory planning

The surgery was performed under local anesthesia, in order to refine targeting in the sensory thalamus using clinical data (i.e., where intraoperative electrical stimulation induced paresthesias overlapping the painful body areas). The target was chosen either in the ventro‐postero‐median nucleus (VPM) or in the ventro‐postero‐lateral (VPL) nucleus, according to the location of the dominant contralateral pain, following the previously described respective stereotactic coordinates of these nuclei^3^ relative to the anterior (AC) and posterior (PC) commissures: *y* = 2–3 mm anterior to CP; vertically at the level of the bi‐commissural plane (*z* = 0). Laterally, the *x* coordinate was chosen according to the topography of the pain: *x* = 12–13 mm (VPM) for facial pain and *x* = 14–15 mm (VPL) for limb pain.^6^, ^11^

Target and trajectory planning were performed on an anatomical preoperative T1 3D SPGR MRI sequence after injection of gadolinium. The optimal position of the electrode was improved with intraoperative electrophysiological exploration using microelectrode recordings (where tactile or proprioceptive stimulation in the painful region induced changes in the discharge pattern of the thalamic neurons) and electrical stimulation,^5^, ^20^ used to ensure that pleasant paresthesias were evoked in the contralateral painful body area and to check the absence of adverse effects related to stimulation, such as muscle contractions, dysarthria, or others.

Once the optimal target was identified, an octopolar lead (Abbott Infinity 8 contact‐directional lead, Chicago, IL, USA) was implanted (target between contacts 2 and 3) and connected to a generator (Abbott Infinity 5 generator). During the postoperative hospital stay, chronic thalamic stimulation was implemented using the contact closest to the optimal target. Stimulation parameters were individually adjusted in order to induce pleasant paresthesias in the painful area: frequency was set between 70 and 130 Hz, pulse width was 120 μs and intensity was set between 0.5 and 1.8 mA across patients. The correct lead placement within the sensory thalamus was confirmed by the postoperative CT scan and the induction of paresthesias in the painful area.

### Clinical data

For the present study which explores the sensory thalamus somatotopy, we collected the clinical data relative to the individual paresthesias induced by the directional monopolar stimulation 1 month after implantation, thus minimizing errors induced by brain edema and microlesion effects, with cingulate stimulation off.^19^ For this mapping, frequency (90 Hz) and pulse width (120 μs) were constant across patients and lead contacts, but the stimulation intensity was set to be the lowest inducing pleasant paresthesias. On a blank layout of the human body, each patient drew the paresthetic body areas he/she felt during stimulation of each directional lead contact.

### Leads reconstruction and atlas projection

For every patient, lead positions were determined on the postoperative 3D CT scan co‐registered with the preoperative 3D T1‐weighted anatomical MRI images using SPM12^21^ and advanced normalization tools.^22^, ^23^ Lead‐DBS toolbox version 2.2.3^24^ was used within Matlab 2016b (The MathWorks, Natick, Massachusetts, USA) for DBS leads visualization and VTA modeling. Normalization to the MNI152 2009b space was also performed by applying ANTS.^23^ The algorithm's accuracy and effectiveness were evaluated elsewhere with SyN.^25^ The lead was then automatically pre‐localized on CT images with the DioDe algorithm^26^, ^27^ and then manually adjusted, if the automatic localization did not match the CT lead artifact (typical adjustments of less than half of the lead's diameter or <0.65 mm).^28^ The Abbott Infinity directional lead has the following characteristics: each contact is 1.5 mm long, with longitudinal spacing of 0.5 mm, and a 1‐3‐3‐1 configuration (Fig. 3A). The two outside contacts (1 and 4) are full rings and perform nondirectional stimulation. At the intermediate levels, contacts 2A, 2B, 2C and 3A, 3B, 3C roughly cover 120° angles, and their stimulation is directional. The electrode orientation was determined on the postoperative CT scan according to the artifact position of a marker located above Contact 4, using lead DBS. The orientation was additionally checked by analyzing the marker location on orthogonal anteroposterior and profile radiographs.

The reconstructed normalized leads were displayed in relation to the thalamic subnuclei of interest represented on the Morel atlas,^29^ namely the VPM, anterior VPL (VPLa) and posterior VPL (VPLp) (Fig. 3B,C).

### Estimation of volumes of tissue activated

Volumes of tissue activated (VTA) were estimated with the FieldTrip‐SimBio FEM pipeline, implemented into the LeadDBS toolbox^30^ using the white matter–gray matter interface defined in the Thalamic DBS Connectivity atlas^31^ and conductivity values for white matter and gray matter of 0.14 and 0.33 S/m, respectively.

After registration on the MNI152 template, the leads and VTAs located in the left thalamus (patients 1, 3, 5 and 6) were flipped to the right side of the brain, in relation to the mid‐sagittal plane, using the ‐*mrflip* option from MRTrix3.^32^ All VTAs were then depicted on the MNI152 template along with the three thalamic subnuclei of interest (Fig. 3), in agreement with the body areas where their stimulation induced paresthesias. The analysis took into account the relationship between the location of VTAs and body paresthesias. This approach was essentially descriptive.

## Results

**Figure 2:**
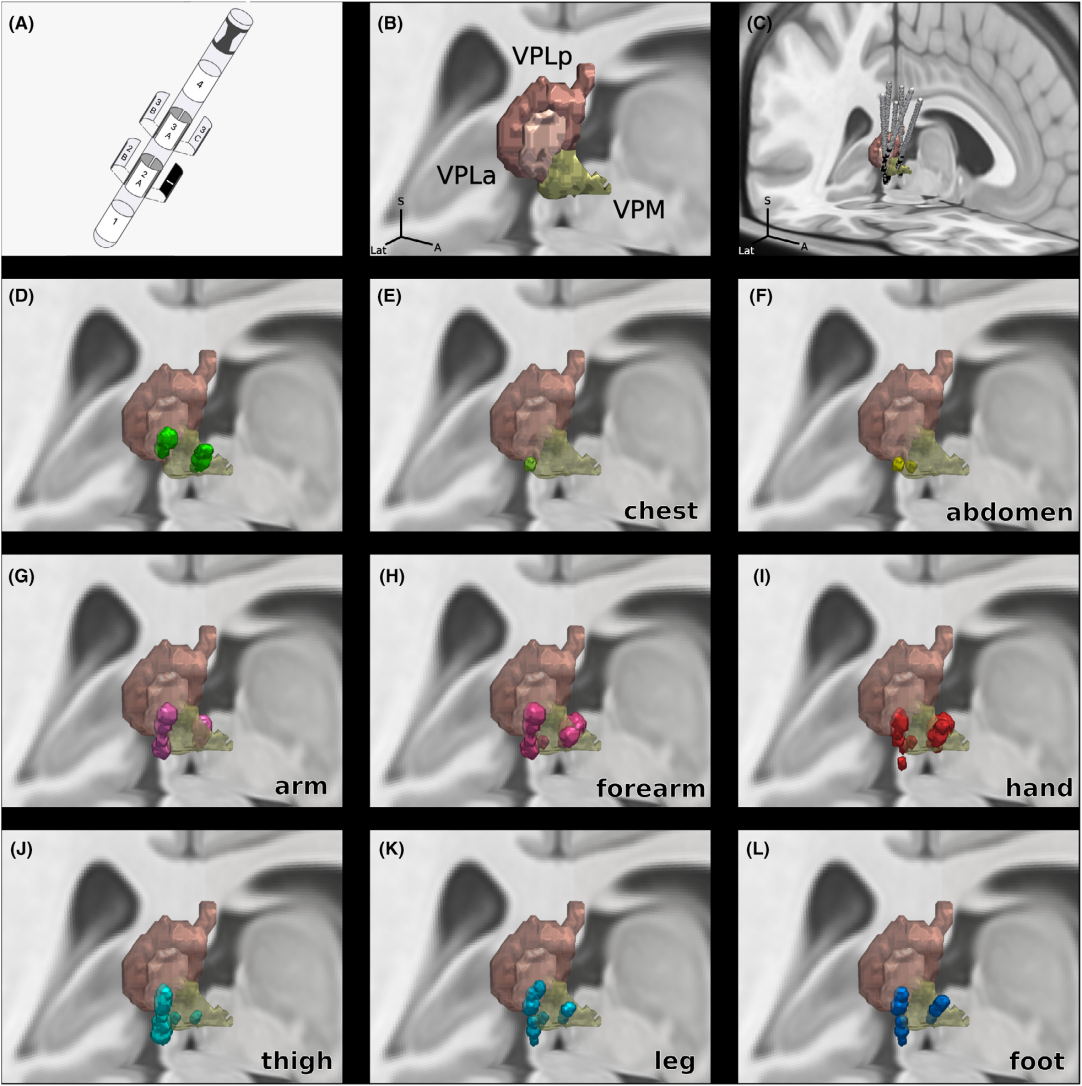
3D representation of volumes of tissue activated (VTA) associated with stimulation‐induced paresthesias in different body areas in six patients treated by sensory thalamic DBS for neuropathic pain. (A) Design of the directional DBS lead. (B) 3D representation of the sensory thalamic subnuclei according to the Morel atlas, from a right superior anterior point of view. (C) Location of the six DBS electrodes. (D–L) Locations of VTAs inducing paresthesias in different body areas. VPLa, ventro‐postero‐lateral anterior nucleus; VPLp, ventro‐postero‐lateral posterior nucleus; VPM, ventro‐postero‐medial nucleus.

All the stimulated contacts induced paresthesias perceived by the patients. All our patients had various degrees of sensory deficits within the painful area. In all of them, stimulation was able to induce paresthesias in the painful area. We did not observe any clear relation between the patients' painful area location, pain duration, sensory disturbances, and somatotopic maps. In each subject, increasing the stimulation amplitude increased the VTA and the surface area where paresthesias were perceived. The patients perceived paresthesias in different body areas depending on the stimulated contact. A single VTA could be associated with paresthesias induced in one or several areas of the body (Fig. 2). Consequently, we recorded 48 distinct paresthesia maps corresponding to 48 distinct VTAs (36 VTAs related to Contacts 2 and 3 directional stimulation and 12 VTAs related to contacts 1 and 4 stimulation), and allowed us to illustrate these projective fields in the sensory thalamus.

**Figure 3:**
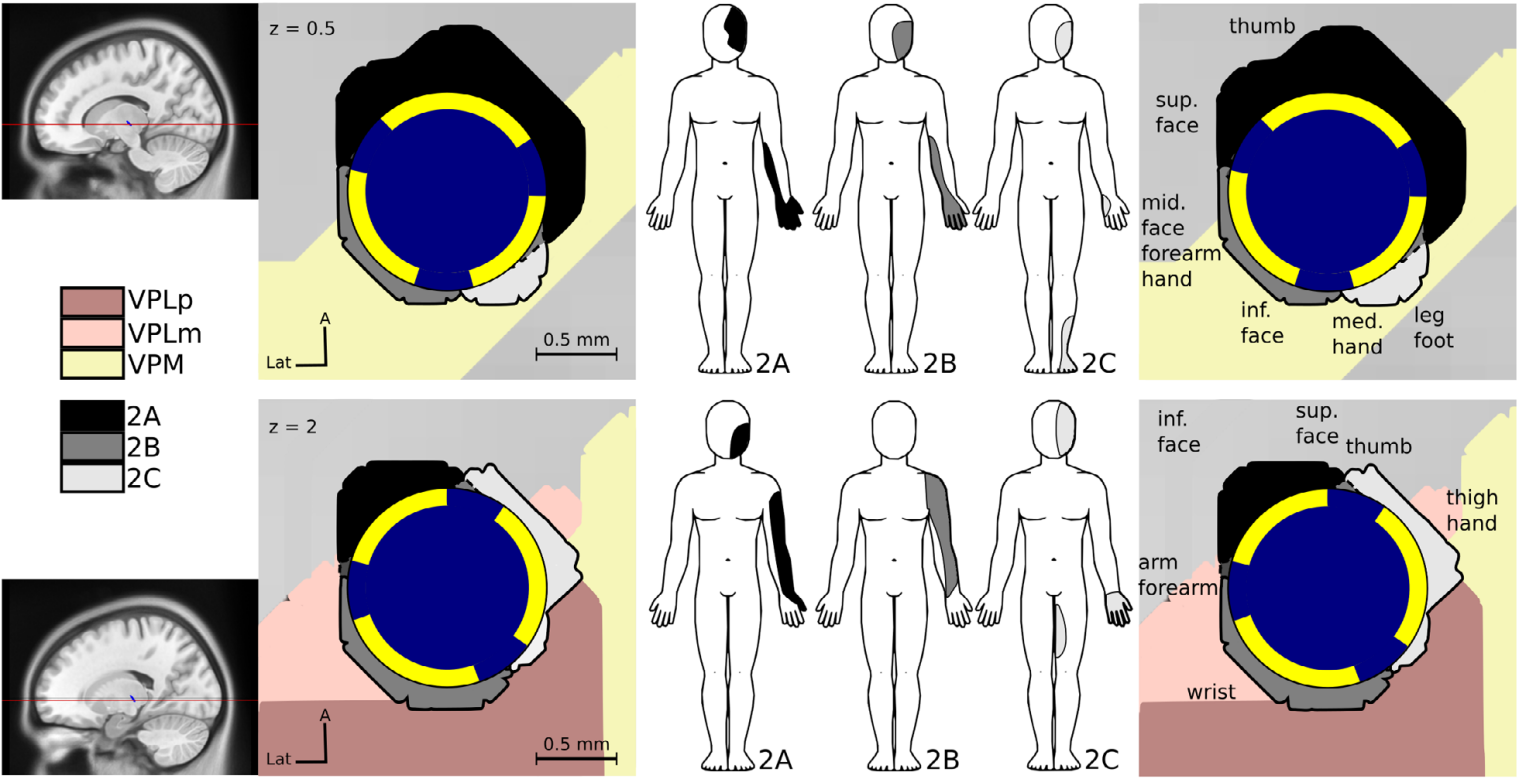
Representation of the paresthesias associated with volumes of tissue activated (VTA) by directional stimulation in Patient 1 (upper row) and Patient 3 (lower row). Electrode insulation is displayed in blue and each of the 3 directional contacts (contacts 2A, 2B, and 2C) are displayed in yellow. VTAs corresponding to lead contacts 2A, 2B, and 2C are displayed respectively in black, gray, and white. Thalamic nuclei are represented in the background: VPLp in brown, VPLm in light brown, and VPM in yellow. For each VTA, the body area where the patient perceived paresthesias is displayed on the body scheme. For example, Patient 1 perceived paresthesias in the head, forearm and hand when contacts 2A and 2B were stimulated, and in head and foot when contact 2C was stimulated. These data suggested that representations of head, upper limb and lower limb were closely located around the electrode, within a distance of 2 mm. Right column displays the individual putative sensory thalamus somatotopic organizations inferred from paresthesias associated with overlapping VTAs in Patients 1 and 3. inf., inferior; sup., superior; VPLa, ventro‐postero‐lateral anterior nucleus; VPLp, ventro‐postero‐lateral posterior nucleus; VPM, ventro‐postero‐medial nucleus.

**Figure 4:**
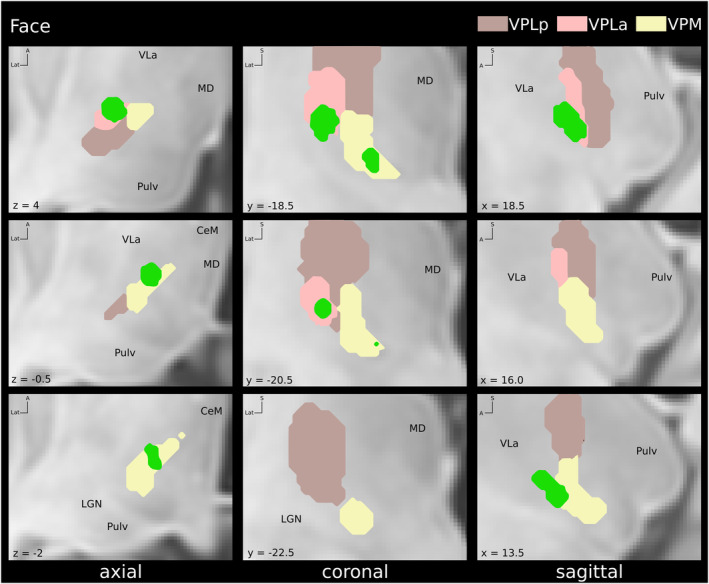
Locations of volumes of tissue activated (VTA) associated with paresthesias in the face within the sensory thalamic nuclei (green). Coordinates are given relative to mid‐commissural point. CeM, central‐medial nucleus; LGN, lateral geniculate nucleus; MD, mediodorsal nucleus; Pulv, pulvinar; VLa, ventral lateral nucleus anterior; VPLa, ventro‐postero‐lateral anterior nucleus; VPLp, ventro‐postero‐lateral posterior nucleus; VPM, ventro‐postero‐medial nucleus.

**Figure 5:**
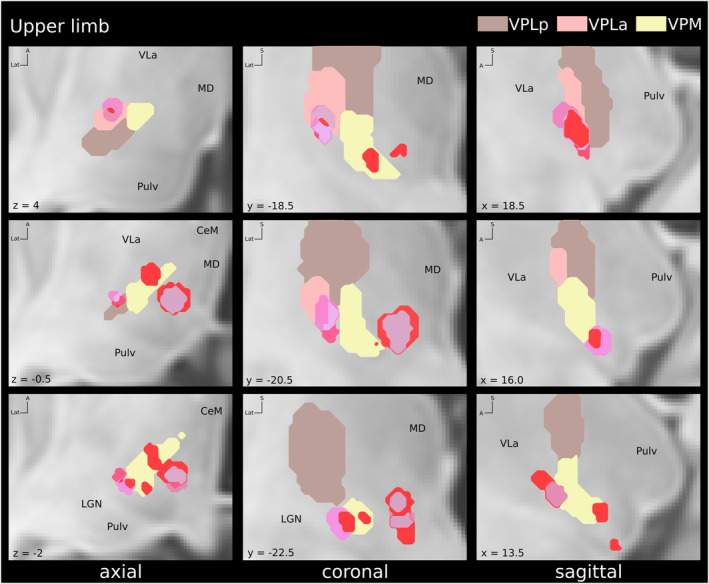
Locations of volumes of tissue activated (VTA) associated with paresthesias in the upper limb within the sensory thalamic nuclei. Pink: shoulder; dark pink: arm; light red: forearm; dark red: hand. Coordinates are given relative to mid‐commissural point. CeM, central‐medial nucleus; LGN, lateral geniculate nucleus; MD, mediodorsal nucleus; Pulv, pulvinar; VLa, ventral‐lateral nucleus anterior; VPLa, ventro‐postero‐lateral anterior nucleus; VPLp, ventro‐postero‐lateral posterior nucleus; VPM, ventro‐postero‐medial nucleus.

**Figure 6:**
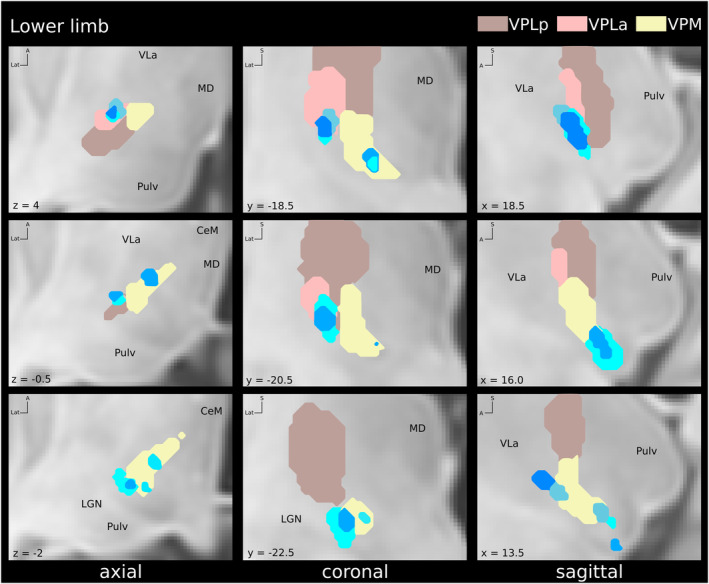
Locations of volumes of tissue activated (VTA) associated with paresthesias in the lower limb within the sensory thalamic nuclei. Light blue = thigh; blue = leg, dark blue = foot. Coordinates are given relative to mid‐commissural point. CeM, central‐medial nucleus; LGN, lateral geniculate nucleus; MD, mediodorsal nucleus; Pulv, pulvinar; VLa, ventral‐lateral nucleus anterior; VPLa, ventro‐postero‐lateral anterior nucleus; VPLp, ventro‐postero‐lateral posterior nucleus; VPM, ventro‐postero‐medial nucleus.

We observed that, in a specific individual, respective body representations of the trunk, upper limb, lower limb, and head were closely located around the lead. Two examples are displayed in Fig. 2. These representations did not follow a common somatotopic organization across patients and were not concordant with the previously described somatotopic organization of the sensory thalamus (Fig. 3). After pooling the data of all the patients, we did not observe a clear somatotopical organization in any direction, and each body area could be located anywhere within the sensory thalamic nuclei (Figs. 3, 4, 5, 6). Moreover, the location of PFs of each body part in the thalamus differed across patients (Fig. 2). There was no difference between central and peripheral neuropathic pain. These results show that, in these patients, the somatotopic organization was not concordant with the classical somatotopic organization of the sensory thalamus previously described.

## Discussion

In patients treated by thalamic directional DBS for chronic neuropathic pain, we observed that DBS‐induced paresthesias were situated at different places on the body for different directions of the stimulation. These DBS‐induced paresthesias frequently affected non‐adjacent body areas, without following a medio‐lateral somatotopic organization and thus challenging the classically accepted somatotopy of the human sensory thalamic nuclei.

The concept of somatotopy of the sensory thalamus has been first described in animals^7^, ^8^, ^33^, ^34^ and then in humans.^11^ Traditionally, the representation of the head and the face is more medial, within the ventro‐posterio‐medial (VPM) nucleus or ventral caudal (Vc) nucleus, according to the nomenclature used, and between 8 and 12 mm lateral to the midline, according to different authors,^35^, ^36^ but may extend up to 18 mm laterally.^6^, ^11^ The thalamic representation of the lower limb is more lateral, classically between 13 and 18 mm lateral to the midline,^11^ but may extend up to 25 mm laterally.^13^ The representation of the upper limb is located in between (Fig. 1). Most of these interpretations are based on intraoperative data obtained in patients with movement disorders treated by DBS or thalamotomies, using microelectrode recordings evoked by sensory stimuli of the contralateral hemibody.^9^, ^10^, ^13^, ^37^, ^38^ Some authors also described the effects of intraoperative stimulation of the sensory thalamus.^10^, ^11^, ^14^, ^16^, ^37^

These pioneer studies were exposed to several biases. Most of them targeted the sensory thalamic nucleus using ventriculography, which does not allow for the accurate localization and visualization of the thalamus, contrary to the fusion of MRI and postoperative CT scan. Moreover, variations in the width of the third ventricle may induce inter‐individual variability of the lateral coordinates of the face or limbs representations within the sensory thalamic nuclei, which may explain the discrepancies of these coordinates across authors. In addition, stimulation intensities that induced paresthesias differed across studies, and no VTA modeling was performed. In our study we used MRI‐based targeting, normalization on a common template and precise localization of the electrodes with postoperative CT, which resulted in a better localization of the VTA within the thalamic sensory nuclei.^11^, ^33^, ^38^, ^39^ Lead trajectories and VTAs were calculated with LeadDBS. The use of LeadDBS (or other software) might lead to inaccuracy. This bias may be tempered by carefully considering the origin and coordinate system and visually checking the data and comparing it to postoperative CT to make sure it is accurate. In addition, our directional electrodes enabled the study of the respective effects of 360° stimulation in individual patients, thus eliminating biases related to the co‐registration of multiple electrodes on a common template. Indeed, in a single patient, even though the electrode was stationary, the paresthesias induced by electrical stimulation covered different body areas depending on the electrically charged directional contact. This observation confirms the concept and strengthens the interest of directional DBS.

Nevertheless, all the pioneer studies were concordant on the somatotopy of the sensory thalamic nuclei, reporting a mediolateral organization of the face, upper limb and lower limb representations, even if mild variations may exist across papers and authors. Importantly, the majority of these data were obtained in patients with movement disorders and without pain.^9^, ^10^, ^11^, ^12^, ^13^ This somatotopy may be different in patients with chronic pain. Indeed, Tasker was the first to mention that in the same patient, the representation of the face and other body regions may coexist at the same site, in a series combining 140 patients with movement disorders and 33 patients with intractable pain.^11^ This suggested a partial overlap of several distinct body areas representations in singular thalamic volumes. In the same team, Davis et al.^16^ compared intraoperative neural responses and thalamic stimulation evoked sensations in 36 chronic pain patients and 24 movement disorders patients without pain. They reported that in pain patients, the projected fields frequently did not match with the receptive fields, in contrast with non‐pain patients whose receptive and projective fields generally matched. They reported that in pain patients, intraoperative thalamic stimulation of a defined site can induce sensations in several nonadjacent body areas, indicating a disorganization of the somatotopy that may be related to neuronal deafferentation. This reorganization may explain the discrepancies observed between the receptive fields, that explore the pathways between the peripheral sensory receptor and the thalamus, and the projective fields (perception of sensory sensations), that reflects the pathways from the thalamus to the sensory cortex. Lesions in the sensory pathway result in sensory loss, changes of the receptive field, deafferentation and neuropathic pain.^16^

Using intraoperative microelectrode recordings, Lenz et al. also reported that the painful areas can be overrepresented within the sensory thalamus.^12^ Based on intraoperative micro‐stimulation, Kiss and Tasker observed an extended thalamic representation of the face in patients suffering from chronic facial pain compared to movement disorders patients. Using a different methodology, namely 3D representation of the volume of thalamic tissue activated by the directional postoperative stimulation through a stationary electrode, our study found similar results, bringing additional arguments in favor of a reorganization of the sensory thalamic nuclei somatotopy in chronic neuropathic pain patients.

Somatotopic reorganizations in sensory relays have been previously reported in animal models of chronic pain^7^, ^34^, ^40^, ^41^, ^42^ and in chronic pain patients,^12^, ^37^, ^38^ either at the cortical level in the primary and secondary sensory cortices or at the thalamic level. Churchill demonstrated in monkeys that thalamic areas corresponding to the deafferented body area following nerve injury, were invaded by representation of adjacent body areas.^40^ Several mechanisms may contribute to this large‐scale sensory reorganization, including rewiring of lemniscal fibers and emergence of ectopic receptive fields.^41^, ^42^ Recently, a study suggested that the somatotopic organization of the thalamocortical structural connectivity is maintained in chronic neuropathic pain patients, which suggested that this functional reorganization more likely involves mechanisms within or before the thalamus.

This somatotopic reorganization has been described in chronic pain syndromes from central or peripheral origin, including complex regional pain syndrome.^16^, ^43^, ^44^ In our study, chronic pain resulted from central and peripheral lesions in four and two cases, respectively.

**Figure 7:**
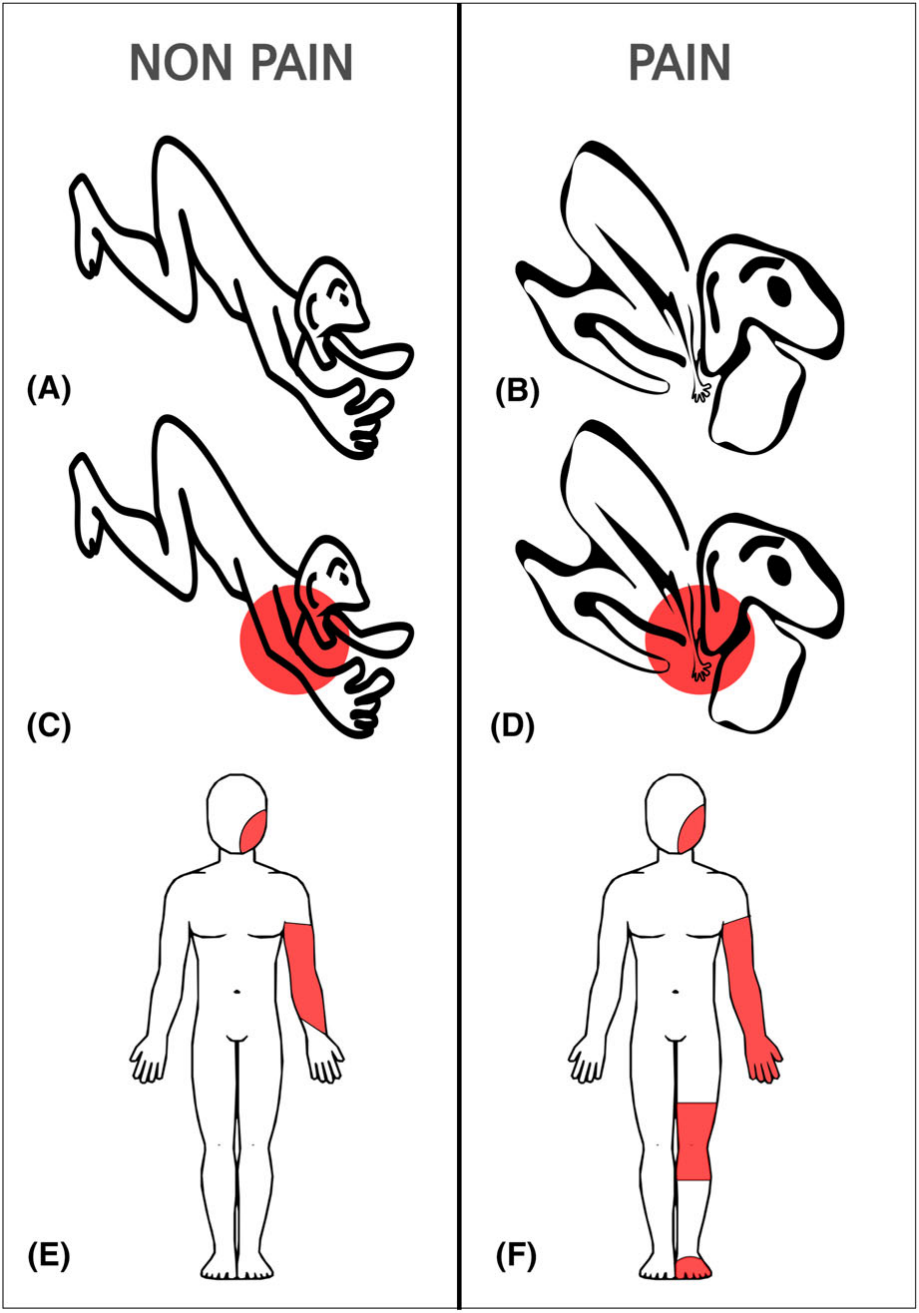
Schematic representation of the thalamic somatotopic reorganization that may occur in patients with neuropathic deafferentation pain. In non‐pain patients, thalamic sensory nuclei are somatotopically organized with face representation medially and lower limb representation laterally (A).^9^ Thalamic stimulation in movement disorder patients (C) induces paresthesias (E) that follows this somatotopic representation. In patients with neuropathic pain, deafferentation may result in expansion of the representation of the adjacent regions into the former representation area of the deafferented region (B). Thalamic stimulation of the same volume of tissue (D) may induce sensations in the regions that overlap the representation of the deafferented region (F).

Deafferentation results in expansion of the representations of the adjacent thalamic regions into the former representation area of the deafferented region (Fig. 7). Consequently, stimulation of this reorganized area can induce sensations in the regions that overlap the deafferented region.

As suggested by our results and previous authors,^11^, ^12^, ^16^ this disorganization is highly variable across subjects and is probably conditional, among other factors, on the topography of the lesion, the sensory loss, the localization of neuropathic pain, pain duration, and the previous individual anatomo‐functional organization, but also on affective consequences of chronic pain.^45^ A multitude of conditions may explain the interindividual variability that we observed in our study concerning the VTAs corresponding to the representations of each body area (Figs. 3, 4, 5, 6). We observed similar reorganizations across patients with central or peripheral deafferentation.

## Conclusion

In chronic neuropathic pain patients treated by thalamic directional DBS, we observed that DBS of the sensory thalamus induced paresthesias in different areas of the body when the direction of stimulation varied. Frequently, these directional DBS‐induced paresthesias were perceived on nonadjacent body areas, were discordant with the classical mediolateral somatotopic organization, thus providing new arguments in favor of a somatotopic disorganization in patients with chronic neuropathic pain of central or peripheral origin.

## Author Contributions

DF, MLM, and AL contributed to the conception and design of the study; DF, AL, AB, JR, and IP contributed to the acquisition of data; DF, AL, and IP contributed to the analysis of data; DF, AL, and IP contributed to drafting the text and preparing the figures.

## Conflict of Interest

DF is consultant for Medtronic, Abbott, and Boston and received research grant from Medtronic and Abbott. MLM is consultant for Medtronic and received research grant from Medtronic. AL received speaker fees from Boston. Other authors have no conflict of interest related to the study.
